# Metformin and Diammonium Glycyrrhizinate Enteric-Coated Capsule versus Metformin Alone versus Diammonium Glycyrrhizinate Enteric-Coated Capsule Alone in Patients with Nonalcoholic Fatty Liver Disease and Type 2 Diabetes Mellitus

**DOI:** 10.1155/2017/8491742

**Published:** 2017-01-04

**Authors:** Rong Zhang, Keran Cheng, Shizan Xu, Sainan Li, Yuqing Zhou, Shunfeng Zhou, Rui Kong, Linqiang Li, Jingjing Li, Jiao Feng, Liwei Wu, Tong Liu, Yujing Xia, Jie Lu, Chuanyong Guo, Yingqun Zhou

**Affiliations:** ^1^Department of Gastroenterology, Shanghai Tenth People's Hospital, Tongji University, School of Medicine, Shanghai 200072, China; ^2^The First Clinical Medical College of Nanjing Medical University, Nanjing 210029, China; ^3^The School of Medicine of Soochow University, Suzhou 215006, China

## Abstract

*Objective*. The present study was conducted to compare the efficacy of metformin combined with diammonium glycyrrhizinate enteric-coated capsule (DGEC) versus metformin alone versus DGEC alone for the treatment of nonalcoholic fatty liver disease (NAFLD) in patients with type 2 diabetes mellitus (T2DM).* Subjects and Methods*. 163 patients with NAFLD and T2DM were enrolled in this 24-week study and were randomized to one of three groups: group 1 was treated with metformin alone; group 2 was treated with DGEC alone; group 3 received metformin plus DGEC combination therapy. Anthropometric parameters, liver function, lipid profile, serum ferritin (SF), metabolic parameters, liver/spleen computed tomography (CT) ratio, and fibroscan value were evaluated at baseline and after 8, 16, and 24 weeks of treatment.* Results*. After 24 weeks, significant improvements in all measured parameters were observed in three groups (*P* < 0.05) except for the improvements in low density lipoprotein cholesterol (LDL-C) and metabolic parameters in group 2 which did not reach statistical significance (*P* > 0.05). Compared with group 1 and group 2, the patients in group 3 had greater reductions in observed parameters apart from CB and TB (*P* < 0.05).* Conclusions*. This study showed that metformin plus DGEC was more effective than metformin alone or DGEC alone in reducing liver enzymes, lipid levels, and metabolic parameters and ameliorating the degree of hepatic fibrosis in patients with NAFLD and T2DM.

## 1. Introduction

Nonalcoholic fatty liver disease (NAFLD) is currently the most common comorbidity and cause of chronic liver disease in adults. The magnitude of the epidemic has indicated that NAFLD is an increasingly recognized public health issue worldwide.

The majority of patients with NAFLD are asymptomatic or have slightly elevated liver enzymes [mild to moderate increase in alanine aminotransferase (ALT) and gamma glutamine transpeptidase (GGT)] or vague upper abdominal pain. NAFLD is characterized by hepatic steatosis during imaging and/or histology and is defined as ≥5%–10% of hepatocytes exhibiting macroscopic steatosis [1, 2] and is diagnosed by no alcohol history or ethanol intake ≤70 g per week in women and ≤140 g per week in men and excludes other etiologies of steatosis, such as virus hepatitis and steatogenic drug administration [3–5]. NAFLD encompasses a clinicopathologic spectrum of conditions ranging from NAFL (simple steatosis, a benign process) to nonalcoholic steatohepatitis (NASH) [2]. Although the pathology of NAFL is usually nonprogressive, some cases may then develop NASH, which is an important risk factor for cerebrovascular disease, cardiovascular disease, and liver fibrosis, and can ultimately progress to liver cirrhosis and even hepatocellular carcinoma [6]. Epidemiological studies have revealed that the prevalence of NAFLD is 20%–40% in western countries, 12%–30% in Asian countries [5], and 20.9% in mainland China [7]. Generally, NAFLD is accompanied by a range of metabolic comorbidities such as visceral obesity, hyperlipidemia, insulin resistance (IR)/diabetes mellitus, and hypertension and is considered a manifestation of the metabolic syndrome (MS) in liver [2, 8]. The prevalence of type 2 diabetes mellitus (T2DM) in patients with NAFLD can reach 70%, and in turn, T2DM increases the risk of NAFLD [9]. With an increase in the rate of urbanization and inappropriate lifestyle changes in modern society, people have a high risk of developing NAFLD due to the growing prevalence of obesity and T2DM [10]. IR is prevalent in NAFLD [11] and is, therefore, a therapeutic target in NAFLD. In addition to treatments aimed at the liver (antioxidants and hepatocyte protective agents), other treatment strategies to ameliorate MS such as improvements in lifestyle, weight loss, and insulin-sensitizing agents (metformin or thiazolidinediones) are necessary [12, 13]. Metformin prescribed in patients with NAFLD and T2DM causes weight loss, reduces liver transaminases, and improves hepatomegaly and IR or insulin secretion [14–16], whereas improvements in histology remain controversial [17, 18]. Thiazolidinediones (rosiglitazone or pioglitazone) have been shown to improve steatosis and liver enzyme levels and regulate glucolipid metabolism. However, the use of thiazolidinediones is restricted due to side effects (ischemic heart disease, heart failure, potential hepatotoxicity, liver failure, weight gain, edema, low bone mineral density, and an increased risk of bladder cancer) [19].

Diammonium glycyrrhizinate enteric-coated capsule (DGEC) is the lipid ligand complex of 18-*α*-diammonium glycyrrhizinate and phosphatidylcholine and has therapeutic effects including a hepatocytic membrane-protective effect, improves liver function, and has a steroid hormone-like effect [20, 21]. However, the steroid hormone-like effect is commonly associated with side effects, especially IR and weight gain. As metformin improves resistance to insulin sensitivity and glucose metabolism, we wondered if metformin combined with DGEC would have a synergistic effect in the amelioration of NAFLD and T2DM. Thus, the present study was designed to assess whether the therapeutic effect of metformin combined with DGEC in NAFLD and T2DM was better than each of these agents alone in patients also treated with diet and exercise.

## 2. Subjects and Methods

### 2.1. Subjects

The study was carried out in Shanghai Tenth People's Hospital (Tongji University, School of Medicine, Shanghai, China), where a cohort of 163 patients aged 18–65 years were recruited from January 2013 to December 2015. Patients diagnosed with NAFLD at ultrasonography (US)/computed tomography (CT) who also had T2DM were eligible for the study. The diagnostic criteria for NAFLD were in accordance with the 2010 Chinese guidelines for the diagnosis and management of NAFLD [3]. The criteria for T2DM were in accordance with the 2013 China guideline for T2DM [22]. Fatty liver was diagnosed by CT before the study, and all patients with a liver/spleen CT ratio <1 were diagnosed with fatty liver. A liver/spleen CT ratio <1.0 and >0.7 was considered mild fatty liver, a liver/spleen CT ratio ≤0.7 and >0.5 was considered moderate fatty liver, and a liver/spleen CT ratio ≤0.5 was considered severe fatty liver.

Exclusion criteria were as follows: (1) Signs of hepatic virus infection (hepatitis B antigen or hepatitis C antibodies), drug-induced hepatic disease, Wilson's disease, cytomegalovirus infection, hemochromatosis, autoimmune liver disease, liver cirrhosis, or liver diseases other than NAFLD; (2) connective tissue diseases or hereditary disorders related to obesity (such as Prader-Willi syndrome) and pathological obesity (such as Cushing syndrome); (3) severe heart disease and/or severe hypertension, severe hepatic and renal dysfunction, cancer, or other severe diseases; (4) history of taking medicine which would disturb observations or the absorption of therapeutic drugs (such as hypotensive drugs, prednisone, amiodarone, and statins); (5) diseases affecting blood glucose levels (such as hyperthyroidism and hypercortisolism) or acute diabetic complications such as ketoacidosis and hyperosmolar coma; (6) poor compliance/adherence to treatment; (7) excessive alcohol intake (ethanol intake >140 g per week in males, >70 g per week in females).

### 2.2. Randomization

Patients (163) were randomly assigned to three groups, and 146 patients completed the study. Group 1 (*n* = 50) was treated with metformin 500 mg three times a day; group 2 (*n* = 50) was treated with DGEC 450 mg three times a day; group 3 (*n* = 46) received metformin (500 mg, three times a day) plus DGEC (450 mg, three times a day). All groups received treatment for 24 weeks, and all enrolled patients were prescribed the same lifestyle modification program (hypocaloric diet in conjunction with regular aerobic exercise; dietary composition: 20% protein, carbohydrate > 50%, and fat < 30% per day; exercise: moderate aerobic physical exercise 60 min per day, at least 5 days a week) during the treatment period. To reduce gastrointestinal side effects, metformin was initially administered at 500 mg per day and was progressively increased to a final dose of 500 mg three times per day. All therapeutic agents were kept stable to prevent possible effects on the study variables.

### 2.3. Observations

At baseline and after 24 weeks of treatment, demographic and anthropometric data (social and family history, physical exam, body weight, and height) were measured in all patients. A blood sample collected after overnight fasting was used to assess liver function [ALT, aspartate aminotransferase (AST), GGT, conjugated bilirubin (CB), and total bilirubin (TB)], lipid metabolism [total cholesterol (TC), triacylglycerol (TG), high-density lipoprotein cholesterol (HDL-C), and low density lipoprotein cholesterol (LDL-C)], serum ferritin (SF), glucose metabolism [fasting blood glucose (FBG), fasting insulin (FINS), homeostasis model assessment of insulin resistance (HOMA-IR)], liver/spleen CT ratio, and the fibroscan value. All patients received CT to measure liver density before and after 24 weeks of treatment. The lipid profile was determined at 4-weekly intervals during treatment. Patients were instructed to limit their alcohol intake during the treatment period, and daily consumption and side effects were reported during follow-up visits. BMI was calculated according to the formula: BMI = weight (in kilograms)/height (in meters) [2]. The homeostatic model assessment of IR (HOMA-IR) was calculated as follows: HOMA-IR = fasting plasma insulin (in mU/L) × FBG (in mmol/L)/22.5. Liver enzymes and lipid profile were measured by a Roche automatic biochemical analyzer (Hitachi Modular P/D, Can 433, Hoffmann-La Roche Inc., Geneva, Switzerland). HbA1c was determined using an automatic glycosylated hemoglobin analyzer (HLC-723G8, TOSOH corporation, Tokyo, Japan). Plasma glucose was measured by an automated glucose oxidase method (Glucose Analyzer 2, Beckman Coulter Inc., Fullerton, California, USA) and insulin was assessed using Roche automatic immunoassay analyzer (Cobas 6000 e601, Hoffmann-La Roche Inc., Geneva, Switzerland). Liver ultrasound was performed in all patients by a single experienced radiologist, blinded to the study, using a 5 MHz Siemens Sonoline Omnia instrument (Siemens Medical Solutions Inc., MountainView, California, USA). The CT value was measured using Siemens Somatom and GE Medical Systems (MX8000, Philips, Cleveland, Ohio, USA). Transient ultrasound elastography (Fibroscan) is a well validated noninvasive tool which measures liver stiffness to quantify liver fibrosis in chronic liver diseases [23]. The fibroscan value was measured using a Fibroscan 502 diasonograph (Echosens, Paris, France). Metformin was produced by the Sino-American Shanghai Squibb Company (Shanghai, China, 500 mg/tablet). DGEC was produced by Jiangsu Chia Tai-Tianqing Pharmaceutical Co., Ltd. (Jiangsu, China, 150 mg/tablet).

### 2.4. Statistical Analysis

All data were analyzed using SPSS version 20.0 software (SPSS, Inc., Chicago, IL, USA) and Prism 6.0 (GraphPad Software Inc., La Jolla, CA, USA) for Windows. Quantitative data were reported as means ± standard deviation (SD). Categorical variables were described using frequency distributions and presented as frequency (%). The Kolmogorov–Smirnov test was used to determine whether the sample data were derived from a normal distribution population. The results, with normal distribution before and after treatment in each group, were evaluated by the paired* t*-test or rank sum test. Intergroup differences between the three groups at baseline and at 24 weeks were compared using one-way analysis of variance or the rank sum test. The Chi-square test was used to compare qualitative variables. All tests were two-sided and a *P* value less than 0.05 was considered statistically significant.

## 3. Results

### 3.1. Patient Characteristics at Baseline

All demographic and biochemical parameters at baseline were similar in the three groups in terms of gender, age, liver function, and metabolic parameters. One hundred and sixty-three patients with NAFLD and T2DM were screened for study participation, and 146 patients (85 males and 61 females) completed the 24-week treatment period. In group 1, 50 completed the study, 31 males (62.0%) and 19 females (38.0%), aged 26–77 years with an average age of 56.30 ± 12.47 years. In group 2, 50 patients completed treatment, 29 males (58.0%) and 21 females (42.0%), aged 29–68 years with an average age of 54.64 ± 9.71 years. In group 3, 4 patients were lost to follow-up and the remaining 25 males (54.3%) and 21 females (45.7%), aged 25–66 years with a mean age of 54.17 ± 10.42 years, completed treatment. The distribution of baseline data was assessed to determine whether the samples followed a normal distribution. At baseline, no statistically significant differences were observed between the three groups with respect to anthropometric parameters (sex, age, weight, and height), BMI, liver function (ALT, AST, and GGT), lipid profile (HbA1c, FBG, and FINS), SF, liver/spleen CT ratio, and fibroscan value (Table 1). The proportion of patients with mild fatty liver at baseline was 29.5% (43 patients; 13 in group 1, 16 in group 2, and 14 in group 3). The proportion of patients with moderate fatty liver at baseline was 44.5% (65 patients; 22 in group 1, 21 in group 2, and 22 in group 3). The proportion of patients with severe fatty liver at baseline was 26.0% (38 patients; 15 in group 1, 13 in group 2, and 10 in group 3) (Table 2).

### 3.2. Anthropometric Parameters after Treatment

Metformin treatment was associated with a significant reduction in weight and BMI before and after 24 weeks of treatment in group 1 (from 76.43 ± 10.16 to 71.27 ± 10.50 kg, *P* < 0.001; from 28.17 ± 2.60 to 26.24 ± 3.52, *P* < 0.001, resp.). Similar results were observed in group 2 (from 75.93 ± 11.17 to 73.28 ± 10.82 kg, *P* < 0.001; from 27.53 ± 2.81 to 26.59 ± 2.95, *P* < 0.001, resp.). Furthermore, compared with group 1 and group 2, group 3 tended to show a more favorable improvement in weight and BMI (*P* < 0.05) (Tables 1 and 3).

### 3.3. Liver Function after Treatment

**Figure 1:**
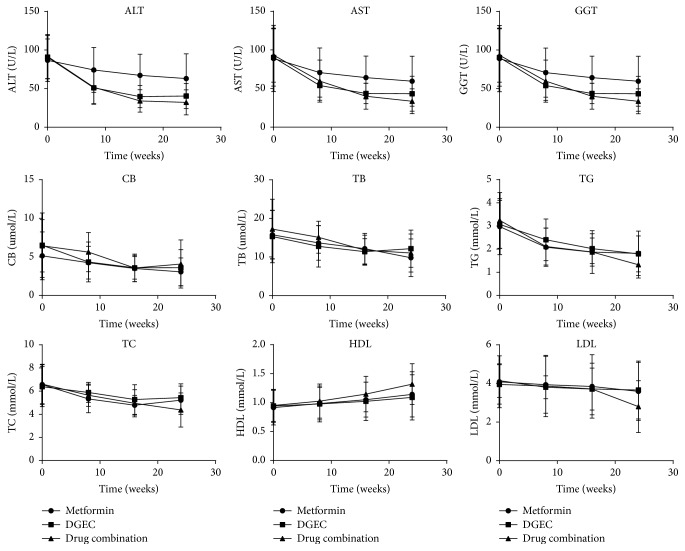
Changes in liver enzymes and lipid parameters over the 24-week treatment period (line chart).

A downward trend in liver function was seen in the three groups (Figure 1). From baseline to the end of the 24-week treatment period, significant reductions in liver enzymes (ALT, AST, and GGT) were observed in the metformin-treated group (*P* < 0.001, *P* = 0.016, and *P* < 0.001, resp.) and in the DGEC-treated group (*P* < 0.001), whereas in the metformin plus DGEC combination group, lower liver enzyme (ALT, AST, and GGT) levels than either the metformin-treated group or the DGEC-treated group were observed at 24 weeks (*P* < 0.001 for group 3 versus group 1 and *P* < 0.05 for group 3 versus group 2). Furthermore, compared with group 1, the mean serum concentrations of liver enzymes in group 2 decreased markedly (*P* < 0.05) (Table 3). As summarized in Table 4 and illustrated in Figure 1, changes in liver enzymes in all three groups were maximal during the first 8 weeks and then gradually declined during the remaining treatment period.

### 3.4. Lipid Parameters after Treatment

**Figure 2:**
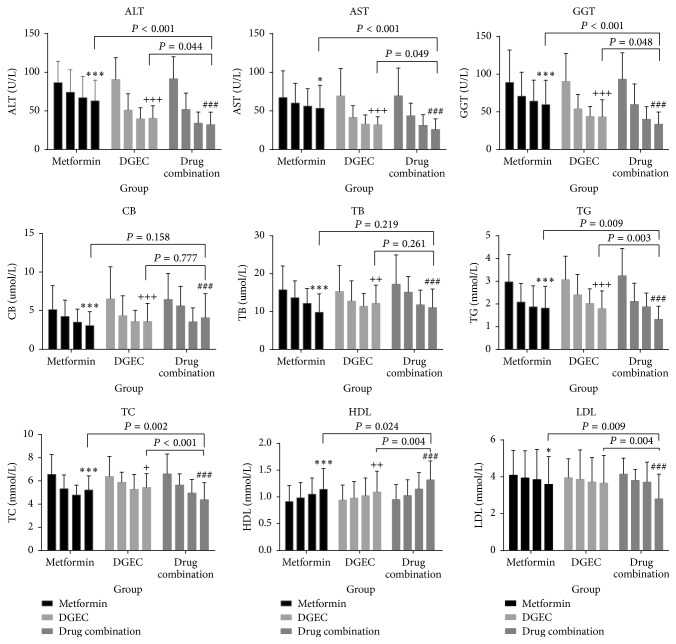
Changes in liver enzymes and lipid parameters over the 24-week treatment period (histogram). *∗∗∗* refer to *P* < 0.001 for comparisons within metformin group. +++ refer to *P* < 0.001 for comparisons within DGEC group. ### refer to *P* < 0.001 for comparisons within drug combination group. *∗* refers to *P* < 0.05 for comparisons within metformin group. ++ refer to *P* < 0.01 for comparisons within DGEC group. + refer to *P* < 0.05 for comparisons within DGEC group.

As shown in Table 3 and Figures 1 and 2, the mean values of TG, TC, and LDL-C declined and HDL-C increased in the three groups. In the metformin-diet-exercise group, despite a significant decrease in the levels of TG (*P* < 0.001), TC (*P* < 0.001), and LDL-C (*P* = 0.047) at 24 weeks, patients had an elevated HDL-C concentration compared with baseline data (*P* < 0.001). The DGEC-diet-exercise group had a reduction in TG (*P* < 0.001), TC (*P* = 0.013), and HDL-C (*P* = 0.004), but a nonsignificant mild reduction in LDL-C (*P* = 0.288) after 24 weeks of treatment. As shown in Table 3, changes in the levels of circulating serum lipids at the final evaluation reached statistical significance in the groups (*P* = 0.004 for TG, *P* < 0.001 for TC, *P* = 0.01 for HDL-C, and *P* = 0.007 for LDL-C). Compared with the metformin-diet-exercise group, statistically significant differences were observed for TG (*P* = 0.009), TC (*P* = 0.002), HDL-C (*P* = 0.024), and LDL-C (*P* = 0.009). In addition, the mean plasma concentrations of TG, TC, HDL-C, and LDL-C were significantly lower in patients in the metformin plus DGEC combination-diet-exercise group than in the DGEC-diet-exercise group (*P* = 0.003, *P* < 0.001, *P* = 0.004, and *P* = 0.004, resp.).

### 3.5. Metabolic Parameters after Treatment

At the end of the 24-week treatment period, HbA1c, FPG, FINS, and HOMA-IR improved in the three groups; however, the changes were significantly different between the groups (*P* < 0.001) (Table 3). In group 1, the patients had decreased mean values of HbA1c (*P* < 0.001), FPG (*P* < 0.001), FINS (*P* = 0.006), and HOMA-IR (*P* = 0.001) after 24 weeks of treatment compared with baseline data. Patients in group 2 had no significant within-group differences in HbA1c, FPG, FINS, and HOMA-IR compared with baseline (*P* = 0.09, *P* = 0.071, *P* = 0.114, and *P* = 0.098, resp.). When the groups were compared at 24 weeks, patients who received metformin plus DGEC had lower HbA1c (*P* = 0.039 for group 3 versus group 1, *P* < 0.001 for group 3 versus group 2, resp.), FPG (*P* = 0.034 for group 3 versus group 1, *P* < 0.001 for group 3 versus group 2), FINS (*P* = 0.007 for group 3 versus group 1, *P* < 0.001 for group 3 versus group 2), and HOMA-IR (*P* = 0.011 for group 3 versus group 1, *P* < 0.001 for group 3 versus group 2) levels than those who received metformin alone or DGEC alone (Table 3).

### 3.6. Other Clinical Characteristics after Treatment

In the metformin-treated group and the DGEC-treated group, reduced levels of SF, liver/spleen CT ratio, and fibroscan value (*P* < 0.001) were observed after 24 weeks of treatment. When the entire cohort at the end of the treatment period was assessed for intergroup comparisons, significant differences were observed for SF, liver/spleen CT ratio, and fibroscan value (*P* < 0.001). At 24 weeks, patients in the drug combination group showed marked improvements in SF, liver/spleen CT ratio, and fibroscan value (*P* < 0.001) compared to the metformin-treated group and DGEC-treated group (Table 3).

### 3.7. Therapeutic Effectiveness

Table 2 shows the number of subjects (percent relative to the baseline, which was considered 100%) in whom improvements were observed after 24 weeks of treatment. Following 24 weeks of treatment, mild fatty liver increased by 28.0% (13 versus 27 patients, before versus after treatment) in group 1, increased by 20.0% (16 versus 26 patients, before versus after treatment) in group 2, and increased by 50.0% (14 versus 37 patients, before versus after treatment) in group 3. Moderate fatty liver was lower in the three groups compared with baseline data (44.0% versus 32.0% in group 1, 42.0% versus 28.0% in group 2, and 47.8% versus 15.2% in group 3). At the end of the study, the number of patients with severe fatty liver in group 3 (4.3%) was lower than that in group 1 (14%) and group 2 (20%).

In general, significant improvements in liver steatosis were observed in the three groups at 24 weeks (*P* < 0.001). Significant improvements, demonstrated by CT, from baseline to 24 weeks, were seen in eight patients (16.0%) in group 1, in seven patients in group 2 (14.0%), and in twelve patients in group 3 (26.1%). A mild improvement in steatosis was observed in 20 patients (40.0%) in the metformin-treated group and in 16 patients (32.0%) in the DGEC-treated group, and significant improvements were seen in 28 patients (60.9%) in the drug combination group. The overall remission rate of fatty liver (including slight and significant improvements) was 87% (40 patients) in group 3 over 24 weeks of treatment, whereas the overall remission rate was 56% and 46% in group 1 and group 2, respectively (Table 5).

### 3.8. Side Effects

No severe side effects were noted during the treatment period, with the exception of a few episodes of mild gastrointestinal disorders (nausea, abdominal pain, and/or diarrhea), fatigue, and an increase in lactate level, which were usually observed early after starting metformin therapy. A marginal increase in lactate level was observed in three patients over the 24-week treatment period, but there were no episodes of acidosis. All patients were able to tolerate treatment with the exception of three patients who discontinued treatment due to a fluctuation in lactate level.

## 4. Discussion

NAFLD is the commonest cause of chronic liver disease worldwide, and NAFLD-associated mortality is rising at an alarming rate, which has attracted worldwide attention. NAFLD is reported to have a high prevalence in subjects with T2DM [2]. NAFLD and T2DM regularly coexist as they share a common pathophysiology, synergistically augment the risk of diabetic complications, and increase the incidence of NAFLD progression to diseases such as cirrhosis and hepatocellular carcinoma [24, 25]. The molecular mechanisms of NAFLD have not yet been entirely clarified but involve the interaction of multiple complex mechanisms. It is currently well recognized that the pathogenesis of NAFLD is due to the so-called two-hit hypothesis, which was initially proposed in 1988 [26]. The first hit refers to hepatic steatosis (the accumulation of triglycerides). Inflammation and oxidative stress caused by oxide metabolites within the hepatocytes are recognized as the second hit [27]. The “multihit” theory has been reported in recent years, which is not in accordance with previous theories, and includes genetic factors, inflammation (especially derived from the gut and adipose tissue), insulin resistance, adipocytokine imbalance, and endoplasmic reticulum stress [6]. However, systemic insulin resistance is still a major determinant in the development of NAFLD independent of coexisting factors [28, 29]. Insulin resistance may compromise the ability of insulin to regulate glucose metabolism. Insulin resistance (adipose tissue and liver) and the associated hyperinsulinemia accelerate the degradation of peripheral adipose tissue and elevate fatty acid level in patients with T2DM. Furthermore, hepatic rather than peripheral insulin sensitivity is independently related to liver fat content [28]. An increase in fatty acid level contributes to more lipoylation to triglycerides, which are deposited in the liver, leading to fatty liver.

Previous studies have indicated that weight loss may have beneficial effects in patients with NAFLD, which may serve as a first-line approach in NAFLD. Exercise was reported to alter the liver mitochondria phospholipidomic profile and maintain mitochondrial function in NASH [30]. A meta-analysis showed that weight loss ≥5% was related to an improvement in hepatic steatosis and weight loss ≥7% improved the NAFLD activity score [31]. However, lifestyle modifications are limited due to difficulties regarding adherence. No treatment strategies targeting patients with NAFLD and T2DM have been established, indicating that etiological treatments (aimed at ameliorating visceral obesity, hyperlipidemia, and diabetes mellitus/insulin resistance) have a high priority. Therefore, insulin-sensitizing agents have been evaluated for the treatment of NAFLD and T2DM, and metformin is an attractive treatment due to its favorable safety profile and potential therapeutic effect [13, 32–34]. It is reported in the literature [35] that metformin not only increases glucose utilization in peripheral tissues, but also inhibits the production of glucose, triglycerides, and cholesterol and stimulates fatty acid oxidation, preventing the progression of NAFLD [18, 36]. However, further studies are needed to determine the exact mechanism of action of metformin in NAFLD.

The dominant effective constituent in DGEC is phosphatidylcholine and glycyrrhizic acid (GA). Phosphatidylcholine is an essential component in cellular membranes and is reported to reduce serum cholesterol level, improve obese status and obesity-related complications, which further decrease the morbidity of NAFLD, and promote the recovery of liver function [37, 38]. GA, a triterpenoid saponin, which is derived from the traditional Chinese medicine, Gancao, was found to modify fatty acids and improve lipid metabolism [39, 40]. Moreover, GA also promotes cell regeneration and was reported to significantly improve hepatocyte steatosis, hepatocyte necrosis, and interstitial inflammation [41, 42]. GA can be hydrolyzed to glycyrrhetinic acid in the human body. It has been reported that the chemical structure of glycyrrhetinic acid is similar to the steroid ring of aldosterone and thereby elevates serum hydrocortisone level and exerts steroid hormone-like effects such as inhibition of synthesis and the release of inflammatory mediators (prostacyclin E2, histamine) [43]. The steroid hormone-like effects of GA and glycyrrhetinic acid also cause some side effects, such as insulin resistance, glucose metabolism disturbance, electrolyte imbalance (hypernatremia and hypokalemia), edema, and weight gain [39].

The present study was undertaken to evaluate the efficacy of metformin plus DGEC in patients with NAFLD and T2DM compared with metformin alone and DGEC alone. Following 6 months of treatment, the overall remission rate in the metformin plus DGEC combination group was better (87%) than that in the metformin-treated group (56%) and the DGEC-treated group (50.9%). Furthermore, the proportion of patients with severe fatty liver decreased from 21.7% to 4.3% in the drug combination group, from 30% to 14% in the metformin-treated group, and from 26% to 20% in the DGEC-treated group. The outcomes in our study indicated that patients treated with metformin plus DGEC may have a more favorable prognosis, and this may be of clinical significance considering the high risk of severe fatty liver progression to NASH, liver fibrosis, and even liver carcinoma. In our study, all treatment groups showed an improvement in anthropometric parameters, liver function, circulating lipid concentrations, metabolic parameters, SF, and fibroscan value. The metformin-treated group tended to have a greater decrease in the lipid profile and metabolic indices compared with the DGEC-treated group, whereas the latter group seemed to have greater improvement in liver enzymes. After 6 months of treatment, patients treated with metformin plus DGEC had greater improvements in weight, liver function, glucolipid metabolism, SF, and liver steatosis than either the metformin alone or DGEC alone group. The findings in the metformin-treated group were generally similar to those observed in previous studies [14, 18, 36, 44], which reported significant improvements in weight loss, glucose and lipid metabolism, and insulin sensitivity. Loomba et al. [18] and Nadeau et al. [14] observed a significant decrease in liver enzymes at 48 weeks and 6 months, respectively. However, the observations for liver enzymes in NAFLD are conflicting. Nair et al. [45] reported that, during the initial 3 months, there was an improvement in ALT, whereas after 3 months of treatment, the concentrations of ALT increased gradually to pretreatment levels. Kazemi et al. [46] attributed the improvements in liver enzymes to weight loss, and metformin had no significant effect on liver enzyme levels. Our outcomes in the DGEC-treated group partly conformed with the results seen in the study by Eu et al. [47] who indicated that GA resulted in statistically significant improvements in FBG, insulin sensitivity, serum free fatty acids, and lipid metabolism in high-fat diet-induced obese rats. Lim et al. [43] also reported similar observations where GA improved dyslipidemia via the selective induction of tissue lipoprotein lipase (a key regulator of lipoprotein metabolism) expression and inhibited the development of insulin sensitivity associated with tissue steatosis. However, we found only a slight decrease in glucose metabolism and insulin sensitivity with no statistical significance, which seemed to contradict previously published studies. Although this result is disheartening, it does not rule out DGEC being potentially efficacious in ameliorating glycolipid metabolism. The inconsistencies in results may be attributed to the weight gain caused by the steroid hormone-like effects, which partly counteracted the benefits of DGEC treatment. However, promising results were obtained when metformin was combined with DGEC, which was more effective than either treatment alone in the management of patients with NAFLD and T2DM. This indicated that metformin may alleviate insulin resistance caused by the steroid hormone-like effects of DGEC, and the combination of metformin and DGEC could have a synergistic effect in ameliorating NAFLD and T2DM.

Our study had the following limitations: (1) In the metformin-diet-exercise-treated group, we could not exclude the benefits of weight loss due to the lack of placebo with lifestyle intervention alone and the lack of an open-label design. (2) Histological data were not available in these patients. (3) It is also possible that 6 months was insufficient time to assess changes in liver function. (4) Sample size was small. (5) Drug dosage was not sufficient to achieve significant effects.

## 5. Conclusions

This study demonstrated that the administration of metformin combined with DGEC was more effective than metformin alone or DGEC alone in improving liver enzymes, glucolipid metabolism, and hepatic steatosis in patients with NAFLD and T2DM. Further randomized, controlled studies with a longer follow-up period are warranted to definitively determine the therapeutic effect of metformin combined with DGEC in patients with NAFLD and T2DM.

## Figures and Tables

**Table 1:** Parameters of patients with T2DM and NAFLD at baseline.

	Group 1 (*n* = 50)		Group 2 (*n* = 50)		Group 3 (*n* = 46)		*P* value^*∗*^
	Baseline	24 weeks	Baseline	24 weeks	Baseline	24 weeks	
Age (yr)	53.46 ± 11.25		54.64 ± 9.71		54.17 ± 10.42		0.85
Gender (M/F)	31/19		29/21		25/21		0.75
Weight (kg)	76.43 ± 10.16	71.27 ± 10.50	75.93 ± 11.17	73.28 ± 10.82	75.92 ± 11.54	66.59 ± 10.28	0.97
Height (m)	1.65 ± 0.08		1.66 ± 0.07		1.65 ± 0.08		0.63
BMI (kg/m^2^)	28.17 ± 2.60	26.34 ± 3.52	27.53 ± 2.81	26.59 ± 2.95	27.96 ± 2.97	24.56 ± 3.03	0.52
ALT (U/L)	86.75 ± 27.59	63.04 ± 26.80	90.79 ± 28.13	40.45 ± 16.30	91.64 ± 28.61	32.23 ± 16.18	0.66
AST (U/L)	67.28 ± 34.68	53.38 ± 29.81	69.38 ± 35.79	32.06 ± 10.41	69.60 ± 36.00	25.91 ± 13.82	0.94
GGT (U/L)	88.96 ± 43.02	59.53 ± 32.38	90.29 ± 37.12	43.32 ± 22.63	93.27 ± 34.98	33.57 ± 16.07	0.86
CB (umol/L)	5.14 ± 3.10	3.07 ± 1.80	6.52 ± 4.18	3.60 ± 2.36	6.45 ± 3.40	4.09 ± 3.12	0.10
TB (umol/L)	15.77 ± 6.29	9.81 ± 4.87	15.31 ± 6.79	12.16 ± 4.83	17.25 ± 7.72	11.04 ± 4.92	0.37
TG (mmol/L)	2.97 ± 1.21	1.82 ± 0.96	3.07 ± 1.04	1.80 ± 0.78	3.24 ± 1.21	1.33 ± 0.57	0.52
TC (mmol/L)	6.57 ± 1.71	5.22 ± 1.22	6.39 ± 1.73	5.44 ± 1.19	6.62 ± 1.71	4.38 ± 1.47	0.79
HDL (mmol/L)	0.91 ± 0.30	1.14 ± 0.39	0.94 ± 0.28	1.09 ± 0.39	0.95 ± 0.28	1.32 ± 0.35	0.80
LDL (mmol/L)	4.09 ± 1.34	3.59 ± 1.50	3.95 ± 1.02	3.66 ± 1.50	4.15 ± 0.87	2.80 ± 1.35	0.68
SF (*µ*g/L)	216.85 ± 81.06	160.18 ± 66.23	223.02 ± 78.37	177.47 ± 67.09	227.50 ± 87.31	130.19 ± 63.63	0.82
HbA1c (%)	8.82 ± 1.79	6.84 ± 1.42	8.70 ± 1.74	8.20 ± 1.90	9.09 ± 1.80	6.23 ± 0.93	0.55
FBG (mmol/L)	9.10 ± 3.02	7.23 ± 1.92	9.27 ± 2.35	8.51 ± 2.22	9.35 ± 2.41	6.38 ± 1.29	0.89
FINS (mU/L)	14.63 ± 3.89	12.04 ± 4.28	15.06 ± 3.71	13.96 ± 3.94	14.84 ± 4.58	9.87 ± 3.39	0.87
HOMA-IR	6.02 ± 3.04	4.16 ± 2.46	6.34 ± 2.51	5.50 ± 2.65	6.56 ± 3.55	2.93 ± 1.51	0.68
CT ratio	0.62 ± 0.16	0.74 ± 0.20	0.62 ± 0.16	0.73 ± 0.21	0.64 ± 0.15	0.89 ± 0.21	0.81
Fibroscan	10.83 ± 2.91	8.54 ± 2.73	11.08 ± 2.33	8.68 ± 2.46	9.94 ± 2.47	6.72 ± 2.02	0.08

*P* values^*∗*^ were calculated by one-factor analysis of variance or the Wilcoxon signed rank test for intergroup comparisons at baseline.

Group 1: metformin-diet-exercise-treated group; group 2: DGEC-diet-exercise-treated group; group 3: drugs combination-diet-exercise-treated group.

**Table 2:** Classification of steatosis in NAFLD before and after treatment.

	Mild		Moderate		Severe		Total
	Before	After	Before	After	Before	After	
Group 1	13 (26.0%)	27 (54.0%)	22 (44.0%)	16 (32.0%)	15 (30.0%)	7 (14.0%)	50 (100%)
Group 2	16 (32.0%)	26 (52.0%)	21 (42.0%)	14 (28.0%)	13 (26.0%)	10 (20.0%)	50 (100%)
Group 3	14 (30.4%)	37 (80.4%)	22 (47.8%)	7 (15.2%)	10 (21.7%)	2 (4.3%)	50 (100%)

**Table 3:** Comparisons within groups and between groups in patients with T2DM and NAFLD at 24 weeks.

	Group 1 (*n* = 50)	Group 2 (*n* = 50)	Group 3 (*n* = 46)	*P* value#	*P*1 value+	*P*2 value+	*P*3 value+
	*P*1 value	*P*2 value	*P*3 value				
Weight (kg)	<0.001	<0.001	<0.001	0.008	0.341	0.032	0.002
BMI (kg/m^2^)	<0.001	<0.001	<0.001	0.004	0.689	0.007	0.002
ALT (U/L)	<0.001	<0.001	<0.001	<0.001	<0.001	<0.001	0.044
AST (U/L)	0.016	<0.001	<0.001	<0.001	<0.001	<0.001	0.049
GGT (U/L)	<0.001	<0.001	<0.001	<0.001	0.014	<0.001	0.048
CB (*µ*mol/L)	<0.001	<0.001	<0.001	0.131	0.495	0.158	0.777
TB (*µ*mol/L)	<0.001	0.008	<0.001	0.058	0.017	0.219	0.261
TG (mmol/L)	<0.001	<0.001	<0.001	0.004	0.999	0.009	0.003
TC (mmol/L)	<0.001	0.013	<0.001	<0.001	0.402	0.002	<0.001
HDL (mmol/L)	<0.001	0.004	<0.001	0.01	0.492	0.024	0.004
LDL (mmol/L)	0.047	0.288	<0.001	0.007	0.804	0.009	0.004
SF (*µ*g/L)	<0.001	<0.001	<0.001	0.002	0.191	0.027	0.001
HbA1c (%)	<0.001	0.09	<0.001	<0.001	<0.001	0.039	<0.001
FBG (mmol/L)	<0.001	0.071	<0.001	<0.001	0.008	0.034	<0.001
FINS (mU/L)	0.006	0.114	<0.001	<0.001	0.015	0.007	<0.001
HOMA-IR	0.001	0.098	<0.001	<0.001	0.030	0.011	<0.001
CT ratio	<0.001	<0.001	<0.001	<0.001	0.912	0.001	<0.001
Fibroscan	<0.001	<0.001	<0.001	<0.001	0.990	0.001	<0.001

*P* value^#^ was calculated by one-factor analysis of variance or the Wilcoxon signed rank test for intergroup comparisons at 24 weeks.

*P*1 value, *P*2 value, and *P*3 value were calculated by the paired *t*-test or the Wilcoxon signed rank test for within groups.

*P*1 value^+^ for comparisons between group 2 and group 1, *P*2 value+ for comparisons between group 3 and group 1, and *P*3 value^+^ for comparisons between group 3 and group 2.

**Table 4:** Liver function and lipid Profile in the three groups at baseline and during treatment.

	Baseline	8 weeks	16 weeks	24 weeks
ALT (U/L)				
Group 1	86.75 ± 27.59	74.22 ± 29.09	67.19 ± 27.40	63.04 ± 26.80
Group 2	90.79 ± 28.13	51.03 ± 21.18	39.70 ± 14.37	40.45 ± 16.30
Group 3	91.64 ± 28.61	51.80 ± 21.22	34.07 ± 14.55	32.23 ± 16.18
AST (U/L)				
Group 1	67.28 ± 34.68	60.19 ± 25.57	56.16 ± 22.66	53.38 ± 29.81
Group 2	69.38 ± 35.79	41.64 ± 15.20	32.98 ± 11.78	32.06 ± 10.41
Group 3	69.60 ± 36.00	43.72 ± 16.35	31.33 ± 13.68	25.91 ± 13.82
GGT (U/L)				
Group 1	88.96 ± 43.02	70.71 ± 31.81	64.23 ± 27.85	59.53 ± 32.38
Group 2	90.29 ± 37.12	53.92 ± 19.09	43.68 ± 13.24	43.32 ± 22.63
Group 3	93.27 ± 34.98	59.70 ± 27.20	40.02 ± 116.75	33.57 ± 16.07
CB (*µ*mol/L)				
Group 1	5.14 ± 3.10	4.26 ± 2.11	3.51 ± 1.70	3.07 ± 1.80
Group 2	6.52 ± 4.18	4.35 ± 2.58	3.59 ± 1.46	3.60 ± 2.36
Group 3	6.45 ± 3.40	5.63 ± 2.54	3.57 ± 1.79	4.09 ± 3.12
TB (*µ*mol/L)				
Group 1	15.77 ± 6.29	13.66 ± 4.46	12.17 ± 3.95	9.81 ± 4.87
Group 2	15.31 ± 6.79	12.76 ± 5.35	11.40 ± 3.36	12.16 ± 4.83
Group 3	17.25 ± 7.72	15.12 ± 4.14	11.78 ± 3.89	11.04 ± 4.92
TG (mmol/L)				
Group 1	2.97 ± 1.21	2.08 ± 0.82	1.87 ± 0.92	1.82 ± 0.96
Group 2	3.07 ± 1.04	2.40 ± 0.90	2.02 ± 0.64	1.80 ± 0.78
Group 3	3.24 ± 1.21	2.12 ± 0.80	1.88 ± 0.61	1.33 ± 0.57
TC (mmol/L)				
Group 1	6.57 ± 1.71	5.33 ± 1.19	4.79 ± 0.85	5.22 ± 1.22
Group 2	6.39 ± 1.73	5.89 ± 0.87	5.28 ± 1.28	5.44 ± 1.19
Group 3	6.62 ± 1.71	5.65 ± 0.96	4.95 ± 1.18	4.38 ± 1.47
HDL-C (mmol/L)				
Group 1	0.91 ± 0.30	0.98 ± 0.28	1.05 ± 0.30	1.14 ± 0.39
Group 2	0.94 ± 0.28	0.98 ± 0.31	1.02 ± 0.33	1.09 ± 0.39
Group 3	0.95 ± 0.28	1.03 ± 0.29	1.15 ± 0.31	1.32 ± 0.35
LDL-C (mmol/L)				
Group 1	4.09 ± 1.34	3.94 ± 1.47	3.85 ± 1.64	3.59 ± 1.50
Group 2	3.95 ± 1.02	3.87 ± 1.59	3.72 ± 1.34	3.66 ± 1.50
Group 3	4.15 ± 0.87	3.81 ± 0.59	3.71 ± 1.09	2.80 ± 1.35

**Table 5:** Therapeutic effectiveness in the three groups.

	Ineffective	Mild efficacy	Significant efficacy	Total
Group 1	22 (44.0%)	20 (40.0%)	8 (16.0%)	50 (100%)
Group 2	27 (54.0%)	16 (32.0%)	7 (14.0%)	50 (100%)
Group 3	6 (13.0%)	28 (60.9%)	12 (26.1%)	46 (100%)
